# Enhancing urothelial carcinoma diagnosis with artificial intelligence–integrated urine cytology: Biopsy‐validated accuracy and efficiency gain

**DOI:** 10.1002/cncy.70110

**Published:** 2026-05-17

**Authors:** Sigfred Lajara, Jacqueline Cuda, Daniel L. Geisler, Caroline Staniszewski, Wei‐Lei Yang, Karen Atkison, Chih‐Yun Lin, Guowei Shao, Tien‐Jen Liu, Barbara Crothers, Rajiv Dhir, Samer N. Khader

**Affiliations:** ^1^ Department of Pathology University of Pittsburgh Medical Center Pittsburgh Pennsylvania USA; ^2^ AIxMed, Inc Santa Clara California USA

**Keywords:** artificial intelligence (AI), digital cytopathology, The Paris System, urine cytology, urothelial carcinoma, whole‐slide image

## Abstract

**Background:**

Urine cytology is a noninvasive tool for detecting urothelial carcinoma, yet its performance depends heavily on expert cytologists and time‐intensive glass slide review. Artificial intelligence (AI)–assisted digital cytology has emerged as a potential solution to improve diagnostic sensitivity and workflow efficiency. This retrospective study aimed to evaluate the diagnostic performance and workflow impact of AIxURO, an AI‐assisted digital urine cytology system, in a high‐volume US medical center.

**Methods:**

Two hundred ThinPrep cytology slides were digitized with two distinct customized scanners integrated into the AIxURO imaging system (AIS‐1 and AIS‐2). Three reviewers independently assessed each case across three diagnostic modalities: microscopy, AIxURO–AIS‐1, and AIxURO–AIS‐2. Performance for binary bladder cancer detection was compared against expert consensus cytology diagnoses; review time, biopsy correlation, and performance in patients presenting with hematuria were also evaluated.

**Results:**

AIxURO (AIS‐1 and AIS‐2) improved diagnostic sensitivity (85.0% and 88.3%) and negative predictive value (NPV) (85.1% and 87.6%) relative to microscopy (79.3% and 82.0%), whereas accuracy remained comparable across modalities. These sensitivity gains were accompanied by lower specificity (85.7% and 82.3% vs. 94.3%) and positive predictive value (PPV) (85.6% and 83.3% vs. 93.3%) compared with microscopy. Both AI modalities reduced classifying atypical urothelial cells and above cases as negative for high‐grade urothelial carcinoma cases, and shortened median review time by 66%–78% (*p* < .0001). Among the 200 cases, 98 (49%) had biopsy confirmation, in which AIxURO showed higher sensitivity and NPV than microscopy. In 16 hematuria cases with biopsy correlation, AI‐assisted screening achieved superior diagnostic performance.

**Conclusions:**

AIxURO enhances sensitivity for detecting urothelial carcinoma and markedly improves screening efficiency, with a tradeoff of reduced specificity/PPV compared with microscopy.

## Abstract 2

Artificial intelligence–assisted digital urine cytology with the AIxURO platform improves urothelial carcinoma detection and reduces false‐negative interpretations while significantly enhancing screening efficiency, at the expense of lower specificity and positive predictive value compared with conventional microscopy, which reflects the expected sensitivity–specificity tradeoff. The platform also demonstrates strong biopsy‐correlated performance, and supports clinical risk stratification in patients presenting with hematuria.

## INTRODUCTION

Urine cytology is essential in bladder cancer evaluation, management, and follow‐up because it is noninvasive, cost‐effective, and highly sensitive in detecting high‐grade urothelial carcinoma (HGUC).^1^ The Paris System for Reporting Urinary Cytology (TPS)^2^ was an effort to standardize the reporting of urine cytology specimens beyond the classic diagnostic categories by capitalizing on the ability of urine cytology to detect HGUC. However, urine cytology comes with inherent factors that can cause subjectivity and difficulty in interpretation in both benign and malignant samples: cellular degeneration, artifacts, suboptimal sample quality, and a wide range of morphologic variations. In addition, urine cytology has long been limited by interobserver variability, particularly in atypical and low‐grade categories, and although TPS has improved standardization, reproducibility remains imperfect across diagnostically challenging categories.^3^, ^4^, ^5^, ^6^, ^7^

Recent national workforce surveys have highlighted a persistent decline in cytologists and pathologists in US medical laboratories, with more than 70%–85% of laboratories reporting shortages driven by reduced training pipelines, increasing case and work complexity, and an aging workforce.^8^, ^9^, ^10^, ^11^ This shrinking workforce poses a growing challenge for high‐volume medical centers and laboratories, and underscores the need for scalable diagnostic support tools.

In response, the cytopathology community has increasingly acknowledged the expanding role of digital cytology and artificial intelligence (AI) in modern diagnostic practice. A recent national survey evaluating current cytotechnology practice identified key professional challenges and areas for improvement.^11^ The survey reported that the responsibilities of cytologists extend beyond conventional cytology to include digital imaging and molecular‐related responsibilities, and approximately half of respondents expressed a positive outlook toward the impact of digital cytology and AI on diagnostic workflows. These findings indicate a growing readiness within the cytology workforce to integrate emerging technologies into routine practice.

Recent years have seen rapid advances in AI‐assisted urine cytology, with platforms ranging from semiautonomous decision‐support algorithms such as AIxURO^12^, ^13^, ^14^, ^15^ and AutoParis‐X,^16^, ^17^ which quantify atypia and highlight suspicious urothelial cells within TPS^2^ framework, to fully automated deep‐learning models capable of whole‐slide classification or prediction of histologic HGUC and recurrence risk.^18^, ^19^ Although these AI tools have demonstrated promising preliminary performance, comprehensive evaluation of their integration into real‐world clinical workflows remains limited. Such validation is essential to determine their true diagnostic value, feasibility, and potential for broad clinical adoption.

In this study, we assessed the performance of the AIxURO platform, an end‐to‐end digital cytology solution with integrated AI analysis and review, in a high‐volume hospital‐based laboratory in the United States. We compared the performance of AIxURO with conventional microscopy, which is the current clinical standard. Prior studies have shown that AIxURO maintains diagnostic accuracy for binary bladder cancer detection while reducing median case review time by 50%–80%.^12^, ^13^, ^14^, ^15^ We hypothesized that similar efficiency gains and maintained diagnostic performance would be observed in our institution. In addition, we explored whether AIxURO can enhance the predictive value of urine cytology when correlated with biopsy outcomes, particularly in patients presenting with hematuria.

## MATERIALS AND METHODS

The study protocol was approved by the Institutional Review Board of the University of Pittsburgh Medical Center (UPMC), Pittsburgh, Pennsylvania (STUDY23100164). We retrospectively and nonconsecutively selected 200 archived, deidentified ThinPrep (Hologic, Marlborough, Massachusetts), nongynecologic voided urine cytology slides. The slides were all Papanicolaou stained, and passed our quality control program. Slides with defects, poor staining, cracks, bubbles, or obstructive debris were excluded. Any annotations and marks on slides were removed. The specimens were obtained from urology patients seen between 2019 and 2023 for clinical indications including unexplained hematuria, urinary tract infections, a history of urothelial carcinoma, or symptoms suggestive of bladder or upper tract urothelial neoplasia. Available related histopathology reports were retrieved. To ensure rigorous and unbiased ground truth assignment, all slides were independently reviewed by two board‐certified and experienced cytopathologists with the diagnostic criteria of TPS.^2^ Slides were excluded if the two cytopathologists disagreed in their interpretations, or if their consensus diagnosis conflicted with the diagnosis documented in the laboratory information system. The consensus diagnoses established the ground truth for subsequent diagnostic accuracy analyses, and consisted of 100 negative for high‐grade urothelial carcinoma (NHGUC) cases, 35 atypical urothelial cells (AUC) cases, 32 suspicious for high‐grade urothelial carcinoma (SHGUC) cases, and 33 HGUC cases.

Before slide scanning, a senior cytologist performed quality control to confirm that each slide was suitable for whole‐slide imaging. Slides were then scanned with two distinct customized digital scanners, hereafter referred to as AIxURO imaging systems AIS‐1 and AIS‐2, which were specifically configured for digital cytology. All slides were scanned at 40× magnification with manual adjustment of the region of interest and focus points. A three‐plane *Z*‐stack scan was performed (best focus *Z* layer ± one layer at 4‐µm intervals), followed by focal‐plane stacking (the three images were then digitally combined “stacked”), to generate a single high‐quality image. This scanning workflow was adapted from previously published digital cytology protocols.^20^, ^21^ After scanning and image stacking, whole‐slide images (WSIs) were generated in each scanner’s proprietary file format. All images were quality checked postimaging to verify optimal focus, contrast, and color fidelity to ensure that they met the quality requirements necessary for subsequent AI algorithm analysis.

**FIGURE 1:**
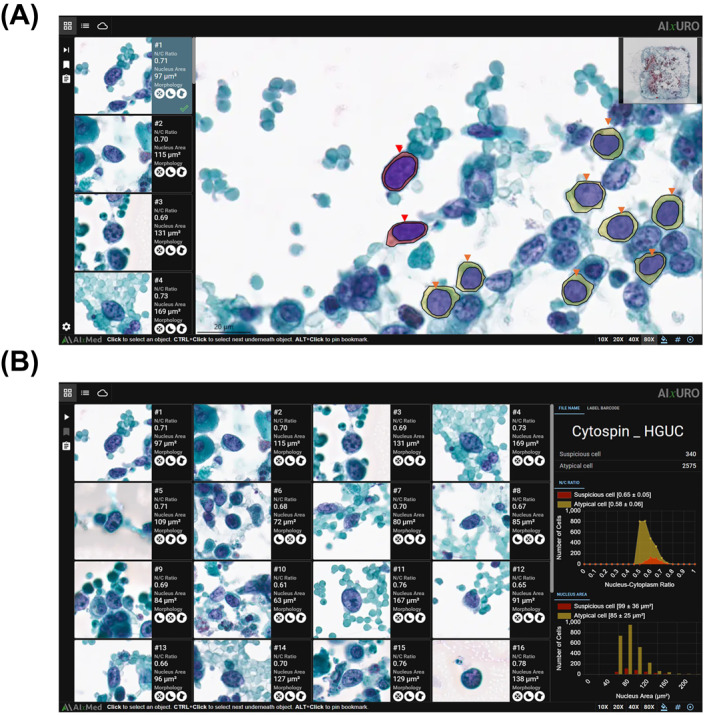
User interface of the AIxURO platform viewer. (A) Dashboard mode: candidate cells identified by the AI algorithm are displayed as thumbnails (*Left*). Selecting a thumbnail automatically navigates the viewer to the corresponding location on the whole‐slide image. Suspicious (high‐risk) urothelial cells identified by AI are highlighted with red arrowheads; atypical (lower risk) cells are highlighted with orange arrowheads. (B) Gallery mode: candidate cells appear in a tiled gallery layout for quick visual assessment. AI indicates artificial intelligence; HGUC, high‐grade urothelial carcinoma; N/C, nucleus‐to‐cytoplasm.

Each quality control–approved WSI was analyzed with the AIxURO platform (AIxMed, Inc, Santa Clara, California), which integrates a deep learning–based instance‐segmentation model within a dedicated viewer interface designed to support urine cytology reporting in accordance with TPS 2.0.^2^ The AIxURO algorithm automatically localizes and categorizes abnormal urothelial cells into suspicious (high cancer risk) and atypical (low cancer risk) groups. For each WSI, it quantifies the total abnormal cell counts in each category, and provides the median nucleus‐to‐cytoplasm ratio and nuclear size displayed in the user interface, the AIxMed Cytology Viewer on desktop computers running Windows 11 Pro with 27‐inch Dell P2725QE monitors (3840 × 2160 resolution; standard refresh rate, 100 Hz). Displays were color calibrated with the Calibrite ColorChecker Display Pro device and Calibrite PROFILER 3.0 software (Figure 1). Notably, the AI classification does not directly equate to TPS categories but provides a probability metric for final diagnosis. These AI‐generated outputs are subsequently reviewed by cytologists and cytopathologists to assign TPS diagnostic categories and render final urine cytology interpretations. The AIxURO algorithm was developed and trained by AIxMed, Inc. The pathology laboratory at the UPMC did not participate in algorithm training or model development, and contributed only deidentified cases and associated clinical/histopathology follow‐up data for validation.

**FIGURE 2:**
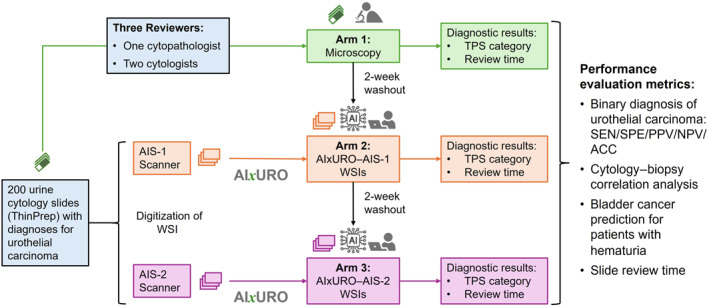
Study flow diagram. Overview of the three‐arm study design comparing conventional microscopy with AI‐assisted digital cytology via the AIxURO platform, including AIS‐1 or AIS‐2 whole‐slide imaging workflows. Diagnostic interpretations were rendered according to TPS, and performance metrics were evaluated across modalities. ACC indicates accuracy; AI, artificial intelligence; NPV, negative predictive value; PPV, positive predictive value; SEN, sensitivity; SPE, specificity; TPS, The Paris System for Reporting Urinary Cytology; WSI, whole‐slide image.

One cytopathologist and two cytologists, blinded to the initial slide diagnosis and ground truth, independently reviewed 200 cases across three study arms separated by a 2‐week washout period. The initial review was performed with manual microscopy of glass slides (arm 1, microscopy). The same reviewers then evaluated AIxURO‐analyzed WSIs acquired with the AIS‐1 scanner (arm 2, AIxURO–AIS‐1) and the AIS‐2 scanner (arm 3, AIxURO–AIS‐2) (Figure 2). Before commencing case review, participants received standardized training courses on the AIxURO platform (viewer navigation, workflow, and interpretation of AI outputs), and completed practice cases to familiarize themselves with the system before timed reads. For each arm, reviewers assigned diagnostic categories in accordance with TPS 2.0 and recorded case review times.

Across the three arms, the reviewers generated a total of 600 diagnostic result pairs. These results were compared against expert consensus cytology diagnoses, which served as the ground truth for this evaluation. The following performance metrics were calculated for binary bladder cancer detection (distinguishing NHGUC from AUC, SHGUC, and HGUC cases): sensitivity, specificity, positive predictive value (PPV), negative predictive value (NPV), and overall accuracy. Review time was also assessed for each diagnostic modality, and defined as the interval from slide evaluation initiation to case sign‐out, with times recorded in seconds for each arm.

The diagnostic performance for each reviewer, as well as aggregated results, was evaluated via sensitivity, specificity, PPV, NPV, and accuracy (agreement with consensus diagnoses), with corresponding case numbers and 95% confidence intervals. Case review times were summarized with medians and interquartile ranges (first and third quartiles). Comparisons of review time to reporting across diagnostic modalities were performed with the Wilcoxon signed‐rank test. All statistical analyses were two sided, with a significance level set at *p* < .05. Data processing and visualization were conducted with R software (version 4.5.0) with the following packages: *dplyr*, *stringr*, *caret*, *ggplot2*, and *ggpubr*.

## RESULTS

There were differences and variability in the agreement among TPS 2.0 categories across the three study arms (Table 1). For NHGUC cases, agreement was highest with microscopy (283 reads; 94.3%) and lower with the AI‐assisted approaches (AIxURO–AIS‐1: 257 reads; 85.7%; AIxURO–AIS‐2: 247 reads; 82.3%). In the AUC category, both AIxURO modalities showed slightly lower agreement (AIS‐1: 48 reads; 45.7%; AIS‐2: 45 reads; 42.9%) relative to microscopy (51 reads; 48.6%). For SHGUC and HGUC, there were differences in the performance of the two AIxURO study arms. AIS‐1 showed lower agreement for SHGUC (29 reads; 30.2%) and HGUC (61 reads; 61.6%) compared with microscopy (33 reads [34.4%] and 72 reads [72.7%], respectively). In contrast, AIS‐2 demonstrated agreement identical to microscopy in both categories (SHGUC: 33 reads; 34.4%; HGUC: 72 reads; 72.7%). Relative to microscopy, the number of AUC consensus reads misclassified as NHGUC decreased from 46 (43.8%) to 43 (41.0%) in AIS‐1 and 33 (31.4%) in AIS‐2; SHGUC reads misclassified as NHGUC decreased from 12 (12.5%) to one (1.0%) and two (2.1%); and HGUC reads misclassified as NHGUC decreased from four (4.0%) to one (1.0%) and zero (0.0%), respectively.

**TABLE 1:** Diagnostic concordance between reviewer interpretation and consensus with The Paris System categories across three modalities.

Reviewer result	Consensus diagnosis	NHGUC (*n* = 300), No. (%)	AUC (*n* = 105), No. (%)	SHGUC (*n* = 96), No. (%)	HGUC (*n* = 99), No. (%)	Total, No.
Arm 1: microscopy (*N* = 600)	NHGUC	283 (94.3)	46 (43.8)	12 (12.5)	4 (4.0)	345
	AUC	15 (5.0)	51 (48.6)	17 (17.7)	12 (12.1)	95
	SHGUC	2 (0.7)	7 (6.7)	33 (34.4)	11 (11.1)	53
	HGUC	0 (0.0)	1 (0.9)	34 (35.4)	72 (72.7)	107
Arm 2: AIxURO–AIS‐1 (*N* = 600)	NHGUC	257 (85.7)	43 (41.0)	1 (1.0)	1 (1.0)	302
	AUC	41 (13.7)	48 (45.7)	33 (34.4)	14 (14.1)	136
	SHGUC	0 (0.0)	8 (7.6)	29 (30.2)	23 (23.2)	60
	HGUC	2 (0.6)	6 (5.7)	33 (34.4)	61 (61.6)	102
Arm 3: AIxURO–AIS‐2 (*N* = 600)	NHGUC	247 (82.3)	33 (31.4)	2 (2.1)	0 (0.0)	282
	AUC	49 (16.3)	45 (42.9)	18 (18.8)	7 (7.1)	119
	SHGUC	1 (0.3)	15 (14.3)	33 (34.4)	20 (20.2)	69
	HGUC	3 (1.0)	12 (11.4)	43 (44.8)	72 (72.7)	130

*Note:* Percentages represent column‐based proportions calculated with the total number of reads in each consensus diagnosis category.

Abbreviations: AUC, atypical urothelial cells; HGUC, high‐grade urothelial carcinoma; NHGUC, negative for high‐grade urothelial carcinoma; SHGUC, suspicious for high‐grade urothelial carcinoma.

For binary bladder cancer detection, TPS 2.0 diagnostic categories were dichotomized into negative (NHGUC; *n* = 100) and positive (AUC+; including AUC, SHGUC, and HGUC; *n* = 100) groups. This approach mirrors contemporary clinical triage practice in urine cytology by identifying patients requiring further evaluation or follow‐up. With consensus cytology as the ground truth (Table 2), both AIxURO modalities demonstrated higher sensitivity and NPV (AIS‐1: 85.0% and 85.1%; AIS‐2: 88.3% and 87.6%) compared with microscopy (79.3% and 82.0%). Conversely, specificity and PPV were lower with AIxURO (AIS‐1: 85.7% and 85.6%; AIS‐2: 82.3% and 83.3%) than with microscopy (94.3% and 93.3%). Overall accuracy remained comparable across modalities (86.8% for microscopy vs. 85.3% for AIxURO arms), which indicated that all three methods delivered similar overall diagnostic performance.

**TABLE 2:** Diagnostic performance across three modalities with consensus cytology as the ground truth.

Modality	Microscopy	AIxURO–AIS‐1	AIxURO–AIS‐2
Sensitivity [cases] (95% CI), %	79.3 [238 of 300] (74.3–83.8)	85.0 [255 of 300] (80.4–88.8)	88.3 [265 of 300] (84.1–91.7)
Specificity [cases] (95% CI), %	94.3 [283 of 300] (91.1–96.7)	85.7 [257 of 300] (81.2–89.4)	82.3 [247 of 300] (77.5–86.5)
PPV [cases] (95% CI), %	93.3 [238 of 255] (89.5–96.1)	85.6 [255 of 298] (81.1–89.4)	83.3 [265 of 318] (78.8–87.3)
NPV [cases] (95% CI), %	82.0 [283 of 345] (77.6–85.9)	85.1 [257 of 302] (80.6–88.9)	87.6 [247 of 282] (83.2–91.2)
Accuracy [cases] (95% CI), %	86.8 [521 of 600] (83.9–89.4)	85.3 [512 of 600] (82.2–88.1)	85.3 [512 of 600] (82.2–88.1)

Abbreviations: CI, confidence interval; NPV, negative predictive value; PPV, positive predictive value.

**FIGURE 3:**
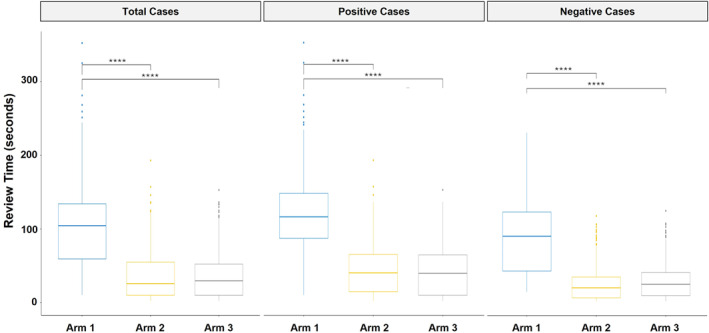
Slide review time across diagnostic modalities. Boxplots show review time distributions (seconds) for all cases, atypical urothelial cells and above cases, and negative for high‐grade urothelial carcinoma cases across microscopy (arm 1, blue color dots/lines), AIxURO–AIS‐1 (arm 2, yellow color dots/lines), and AIxURO–AIS‐2 (arm 3, gray color dots/lines). Both AI‐assisted modalities significantly reduced median review times compared with microscopy across all groups (****p < .0001). AI indicates artificial intelligence.

Slide review time (SRT) was compared across the three diagnostic modalities. As shown in Figure 3, both AIxURO workflows were associated with significantly shorter median SRTs than conventional microscopy across all cases. The median SRT for microscopy was 104 s, compared with 26 s in AIxURO–AIS‐1 (−75.0%) and 29 s in AIxURO–AIS‐2 (−72.1%) (both *p* < .0001). This substantial reduction in SRT was observed in both AUC+ and NHGUC subgroups. Among AUC+ cases, the median SRT was 116 s with microscopy, whereas AIxURO modalities demonstrated significantly shorter median SRTs of 40 s (−65.5%; *p* < .0001). Similarly, for NHGUC cases, microscopy had a median SRT of 90 s, whereas AIxURO‐assisted review reduced median SRTs to 20 s in AIS‐1 (−77.8%) and 25 s in AIS‐2 (−72.2%) (both *p* < .0001).

Of the 200 urine cytology cases, 98 (49.0%) had available follow‐up histopathology (Table 3). We restricted our analysis to 72 cases (36.0%) with biopsies obtained within 3 months of urine collection. With biopsy diagnosis as the ground truth, negative cases included NHGUC, low‐grade urothelial carcinoma, and AUC (favor reactive) (*n* = 18), whereas positive cases included AUC (not otherwise specified), SHGUC, urothelial carcinoma in situ, and HGUC (*n* = 54). As shown in Table 4, both AIxURO modalities demonstrated higher sensitivity and NPV (AIS‐1: 92.0% and 69.8%; AIS‐2: 93.2% and 71.1%) compared with microscopy (84.6% and 56.1%). Specificity and PPV were lower for the AIxURO modalities (AIS‐1: 55.6% and 86.1%; AIS‐2: 50.0% and 84.8%) than for microscopy (59.3% and 86.2%). Overall accuracy was modestly higher with AIxURO (82.9% for AIS‐1; 82.4% for AIS‐2) compared with microscopy (78.2%), which indicated that the AI‐assisted modalities improved overall diagnostic performance relative to microscopy.

**TABLE 3:** Distribution of biopsy diagnoses and definition of biopsy‐correlated subgroups (overall and hematuria).

Cases with biopsy reports (*n* = 98; 49.0%)	Cases, No. (%)	Cytology–biopsy correlation, ≤3 months (*n* = 72; 36.0%)	Cases, No. (%)	Cytology–biopsy correlation, ≤3 months, in hematuria cases (*n* = 16; 8.0%)	Cases, No. (%)
NHGUC	20 (20.4)	NHGUC	14 (19.4)	NHGUC	5 (31.3)
LGUC	5 (5.1)	LGUC	3 (4.2)	LGUC	1 (6.3)
AUC (favor reactive)	4 (4.1)	AUC (favor reactive)	1 (1.4)	AUC (favor reactive)	0 (0.0)
AUC	2 (2.0)	AUC	1 (1.4)	AUC	0 (0.0)
SHGUC	1 (1.0)	SHGUC	1 (1.4)	SHGUC	0 (0.0)
CIS	17 (17.3)	CIS	14 (19.4)	CIS	1 (6.3)
HGUC	49 (50.0)	HGUC	38 (52.8)	HGUC	9 (56.3)

*Note:* Diagnostic performance for the ≤3‐month biopsy correlation subgroup and the hematuria subgroup is summarized in Tables 4 and 5, respectively.

Abbreviations: AUC, atypical urothelial cells; CIS, carcinoma in situ; HGUC, high‐grade urothelial carcinoma; LGUC, low‐grade urothelial carcinoma; NHGUC, negative for high‐grade urothelial carcinoma; SHGUC, suspicious for high‐grade urothelial carcinoma.

**TABLE 4:** Diagnostic performance across three modalities with biopsy reports as the ground truth.

Modality	Microscopy	AIxURO–AIS‐1	AIxURO–AIS‐2
Sensitivity [cases] (95% CI), %	84.6 [137 of 162] (78.1–89.8)	92.0 [149 of 162] (86.7–95.7)	93.2 [151 of 162] (88.2–96.6)
Specificity [cases] (95% CI), %	59.3 [32 of 54] (45.0–72.4)	55.6 [30 of 54] (41.4–69.1)	50.0 [27 of 54] (36.1–63.9)
PPV [cases] (95% CI), %	86.2 [137 of 159] (79.8–91.1)	86.1 [149 of 173] (80.1–90.9)	84.8 [151 of 178] (78.7–89.8)
NPV [cases] (95% CI), %	56.1 [32 of 57] (42.4–69.3)	69.8 [30 of 43] (53.9–82.8)	71.1 [27 of 38] (54.1–84.6)
Accuracy [cases] (95% CI), %	78.2 [169 of 216] (72.1–83.6)	82.9 [179 of 216] (77.2–87.6)	82.4 [178 of 216] (76.7–87.2)

Abbreviations: CI, confidence interval; NPV, negative predictive value; PPV, positive predictive value.

As shown in Table 3, 16 cases (8.0%) displayed hematuria, of which six were biopsy negative and 10 were biopsy positive within 3 months of urine collection. With biopsy diagnosis as the ground truth, both AIxURO modalities demonstrated improved diagnostic performance compared with microscopy (Table 5). Sensitivity, PPV, and NPV were higher in AIxURO–AIS‐1 (96.7%, 87.9%, and 93.3%) and AIxURO–AIS‐2 (100.0%, 88.2%, and 100.0%) relative to microscopy (90.0%, 87.1%, and 82.4%). Specificity remained comparable across all modalities (77.8%). Overall accuracy was also higher with AIxURO (89.6% for AIS‐1; 91.7% for AIS‐2) than with microscopy (85.4%).

**TABLE 5:** Diagnostic accuracy in hematuria cases across three modalities relative to biopsy findings.

Modality	Microscopy	AIxURO–AIS‐1	AIxURO–AIS‐2
Sensitivity [cases] (95% CI), %	90.0 [27 of 30] (73.5–97.9)	96.7 [29 of 30] (82.8–99.9)	100.0 [30 of 30] (88.4–100.0)
Specificity [cases] (95% CI), %	77.8 [14 of 18] (52.4–93.6)	77.8 [14 of 18] (52.4–93.6)	77.8 [14 of 18] (52.4–93.6)
PPV [cases] (95% CI), %	87.1 [27 of 31] (70.2–96.4)	87.9 [29 of 33] (71.8–96.6)	88.2 [30 of 34] (72.5–96.7)
NPV [cases] (95% CI), %	82.4 [14 of 17] (56.6–96.2)	93.3 [14 of 15] (68.1–99.8)	100.0 [14 of 14] (76.8–100.0)
Accuracy [cases] (95% CI), %	85.4 [41 of 48] (72.2–93.9)	89.6 [43 of 48] (77.3–96.5)	91.7 [44 of 48] (80.0–97.7)

Abbreviations: CI, confidence interval; NPV, negative predictive value; PPV, positive predictive value.

## DISCUSSION

Overall, our results closely mirror those reported in a prior clinical study from Taiwan that used the AIxURO platform.^14^ The reproducibility of performance across geographically distinct practice settings supports the potential role of AIxURO as a scalable solution to workforce shortages and increasing workload demands in cytopathology laboratories. In the overall cohort, with consensus cytology diagnoses as the ground truth, AIxURO demonstrated improved bladder cancer detection compared with manual microscopy, reflected by increased sensitivity, albeit with a concomitant reduction in specificity (Table 2). This tradeoff is noteworthy, given that conventional urine cytology has historically prioritized specificity over sensitivity. Similarly, in the subset of cases with biopsy confirmation as the ground truth, AIxURO achieved higher sensitivity and NPV than microscopy, and showed improved concordance with follow‐up histology (Table 4). In addition, AIxURO significantly reduced the median SRT by 66%–78% across all diagnostic categories (Figure 3). Both the increased sensitivity and better review efficiency are factors that would benefit different clinical workflows. However, the decreased specificity could indicate an increase in unnecessary follow‐up procedures. Notably, the AIxURO methods reduced the number of abnormal TPS categories (AUC, SHGUC, and HGUC) that were reviewed as NHGUC, which thereby enhanced sensitivity for AUC+ diagnoses.

The improved sensitivity observed with AIxURO was accompanied by lower specificity and PPV, which reflects an expected sensitivity–specificity tradeoff in urine cytology triage. This likely derives from AI‐assisted review prioritizing candidate atypical/suspicious cells, which can lower the effective threshold for calling AUC+ in borderline/reactive cases and increase NHGUC‐to‐AUC reclassification. Apparent “false positives” may also be amplified by reference‐standard constraints, including consensus cytology as ground truth and incomplete verification of true negatives. Finally, study conditions, such as limited clinical context, deviation from the laboratory’s routine cytologist‐to‐pathologist workflow, and a potential learning curve with AI‐assisted review, may further bias interpretations toward conservative AUC+ assignments.

Because the system is not limited to a specific proprietary scanner, it can be integrated into different types of laboratories and clinical workflows. The two different customized scanners in the AIxURO imaging system produced comparable results (AIxURO–AIS‐1 and AIxURO–AIS‐2 arms). When considered alongside the prior Taiwan study, which used an Aperio AT2 scanner (Leica Biosystems, Wetzlar, Germany), the consistent performance across various scanner types suggests that the AIxURO platform is robust and can handle variations in whole‐slide imaging systems.

The agreement in NHGUC and AUC categories was lower with AIxURO than with microscopy. However, both AI modalities significantly reduced the number of higher risk cases, particularly SHGUC, which were downgraded to NHGUC, and thereby increased sensitivity. This is particularly important because the downgrade from SHGUC to NHGUC can lead to drastically different management and follow‐up, especially in the initial presentation of patients. Notably, AIS‐2 achieved SHGUC and HGUC agreement identical to microscopy, which suggests the reliability of AI‐assisted categorization across scanners. The increase in accuracy and sensitivity may improve risk stratification and disease detection, especially for AUC/SHGUC categories, by reducing missed cases and enabling earlier intervention. A higher NPV may reduce unnecessary invasive procedures, which lowers patient burden and health care costs.

The hematuria subset provides additional insight into the potential clinical utility of AI‐assisted urine cytology in triage settings. Although hematuria is a common presentation of bladder cancer, it can also be seen in benign conditions, such as urinary tract stones, trauma, and infection. In a post hoc, exploratory analysis of hematuria cases with biopsy correlation, AIxURO demonstrated higher sensitivity, PPV, NPV, and overall accuracy than conventional microscopy while maintaining comparable specificity (Table 5). These findings suggest that AI‐assisted digital cytology may improve detection and help triage high‐risk individuals for cystoscopy, which potentially reduces unnecessary procedures or follow‐up visits for low‐risk cases. However, given the small sample size, these results should be considered hypothesis generating, and require validation in larger, prospectively designed cohorts.

Review time data (Figure 3) highlight the significant efficiency gains with AI, which showed an overall reduction in case review time by more than 60%. Across all modalities, positive cases (AUC+) required longer review times than negative cases, consistent with the increased diagnostic complexity of abnormal specimens.

There are several limitations in this study. Reviewers were provided only patient age and gender, whereas in routine practice, cytologists and pathologists typically have access to more clinical information, including cystoscopy findings and relevant medical history. In this data set, clinical history was limited to what was documented in the cytology report. Also, the pathologist reviewer was not provided with the cytologist’s interpretation before review of the glass slide or AI‐assisted images, which is in contrast to the practice in this laboratory. Introduction of new technologies, such as AI‐assisted image review, is typically associated with a learning curve. Concordance for SHGUC/HGUC improved between arms 2 and 3, which possibly reflects greater familiarity with the interpretive use of AI. This was the first experience with AIxURO for the investigators, and subsequent exposure may improve concordance. Although TPS 2.0 is being used in our practice and familiarity with TPS 2.0 was ensured at the beginning of the study, strict adherence to the criteria was not monitored or enforced. The ground truth used for urine cytology in this study is inherently imperfect, given that diagnostically challenging cases that failed to achieve expert consensus were excluded. Unfortunately, there are no means of confirming that NHGUC cases are true negatives. In fact, the reassignment of some of these cases to AUC (microscopy: 15 reads; AIxURO–AIS‐1: 41 reads; AIxURO–AIS‐2: 49 reads) (Table 1) suggests that there may be cytologic features that were previously missed but captured on further review. The use of follow‐up histology as ground truth is also less than ideal because the decision to perform a biopsy is not solely based on cytology results, especially in NHGUC and AUC cases. Because an AUC diagnosis could shorten the follow‐up interval or necessitate additional work‐up, it was included in the positive group for statistical analysis. The biopsy outcomes in this study are based solely on pathology report review rather than review of biopsy slides. Finally, the relatively small number of cases with biopsy follow‐up and hematuria presentation, along with the limited number of reviewers, restricts the strength and generalizability of our conclusions. We are continuing to conduct a larger scale study with expanded case numbers, additional reviewers, and full access to clinical information to validate and further support the findings of this work.

In summary, this study represents the first US clinical experience evaluating the performance of the AIxURO platform in a high‐volume medical center and its application to urine cytology in comparison with conventional microscopy. Assessment across multiple whole‐slide imaging systems allowed the evaluation of performance consistency and variability across digital cytology environments. It demonstrated strong diagnostic performance in urine cytology in comparison with conventional microscopy. Importantly, this work also provides the first biopsy‐confirmed assessment of AIxURO as a diagnostic tool, which reinforces its clinical validity against histopathologic outcomes. Finally, our analysis of patients presenting with hematuria highlights the potential use of AIxURO for risk stratification and bladder cancer triage by offering a practical pathway to streamline evaluation and prioritize cystoscopy in real‐world practice.

## AUTHOR CONTRIBUTIONS

**Sigfred Lajara:** Writing—review and editing, writing—original draft, conceptualization, investigation, methodology, and validation. **Jacqueline Cuda:** Methodology, investigation, and data curation. **Daniel L. Geisler:** Methodology, investigation, and data curation. **Caroline Staniszewski:** Methodology, investigation, and data curation. **Wei‐Lei Yang:** Writing—original draft, writing—review and editing, project administration, investigation, validation, methodology, data curation, formal analysis, and conceptualization. **Karen Atkison:** Software, methodology, writing—original draft, and writing—review and editing. **Chih‐Yun Lin:** Project administration and software. **Guowei Shao:** Writing—original draft, formal analysis, data curation, and visualization. **Tien‐Jen Liu:** Supervision, resources, writing—review and editing, funding acquisition, conceptualization, and investigation. **Barbara Crothers:** Writing—review and editing, writing—original draft, supervision, conceptualization, investigation, validation, and methodology. **Rajiv Dhir:** Supervision, writing—review and editing, and resources. **Samer N. Khader:** Supervision, resources, writing—review and editing, writing—original draft, conceptualization, and investigation.

## CONFLICT OF INTEREST STATEMENT

Wei‐Lei Yang, Karen Atkison, Chih‐Yun Lin, Guowei Shao, Tien‐Jen Liu, and Barbara Crothers are employees of AIxMed, Inc. Wei‐Lei Yang reports owning stock in AIxMed, Inc. Chih‐Yun Lin reports owning stock in AIxMed, Inc. Tien‐Jen Liu reports receiving travel support from and holding patents with AIxMed, Inc. The other authors declare no conflicts of interest.
